# Niedrige Inzidenz von SARS-CoV‑2-Infektionen bei Krankenhausmitarbeitern eines Maximalversorgers: Ergebnisse einer prospektiven serologischen Kohortenstudie der ersten und zweiten COVID‑19-Welle

**DOI:** 10.1007/s00063-021-00890-5

**Published:** 2022-01-03

**Authors:** Julian Hupf, Ralph Burkhardt, André Gessner, Constantin Maier-Stocker, Markus Zimmermann, Frank Hanses, David Peterhoff

**Affiliations:** 1 Zentrale Notaufnahme, Universitätsklinikum Regensburg, 93053 Regensburg, Deutschland; 2 Institut für Klinische Chemie und Laboratoriumsmedizin, Universitätsklinikum Regensburg, Regensburg, Deutschland; 3 Institut für klinische Mikrobiologie und Hygiene, Universitätsklinikum Regensburg, Regensburg, Deutschland; 4 Abteilung für Krankenhaushygiene und Infektiologie, Universitätsklinikum Regensburg, Regensburg, Deutschland; 5 Institut für medizinische Mikrobiologie und Hygiene, Universität Regensburg, Regensburg, Deutschland

**Keywords:** COVID‑19, Krankenhauspersonal, Seroprävalenz, Antikörperstatus, SARS-CoV‑2, COVID‑19, Hospital personnel, Seroprevalence, Antibody status, SARS-CoV‑2

## Abstract

**Hintergrund:**

Mitarbeiter im Gesundheitswesen mit Kontakt zu COVID‑19-Patienten sind einem erhöhten Risiko einer Infektion mit SARS-CoV‑2 ausgesetzt. Ziel dieser seroepidemiologischen Studie war es, das Infektionsrisiko für Klinikmitarbeiter eines Maximalversorgers zu evaluieren.

**Methodik:**

Im Rahmen einer prospektiven Kohortenstudie wurden von März bis Juli 2020 (1. Welle) bei unmittelbar in der Versorgung von COVID‑19-Patienten eingesetzten Mitarbeitern im Abstand von jeweils 2 Wochen serologische Untersuchungen auf Antikörper gegen SARS-CoV‑2 durchgeführt. Von Dezember 2020 bis Februar 2021 (2. Welle) fand eine erneute Untersuchung des Antikörperstatus statt.

**Ergebnisse:**

Die Seroprävalenz von Antikörpern gegen SARS-CoV‑2 betrug am Studienende im Februar 2021 5,1 %. Die kumulative Inzidenz betrug nach einer medianen Beobachtungsdauer von 261 Tagen 3,9 %.

**Schlussfolgerung:**

In der untersuchten Kohorte von Klinikmitarbeitern, die in der Akutversorgung von COVID‑19-Patienten eingesetzt werden, fand sich unter den angewandten Hygiene- und Schutzmaßnahmen ein niedriges und mit der Gesamtbevölkerung vergleichbares Risiko einer SARS-CoV-2-Infektion.

## Abstract (en)

**Background:**

Healthcare workers caring for coronavirus disease 2019 (COVID‑19) patients are at an increased risk for a severe acute respiratory syndrome coronavirus 2 (SARS-CoV-2) infection. The aim of this seroepidemiological study was to evaluate the risk of infection for employees at a tertiary care hospital.

**Methods:**

Serological tests for antibodies against SARS-CoV‑2 were carried out in a prospective cohort of employees directly involved in the care of COVID‑19 patients every 2 weeks from March to July 2020 (1st wave). Antibody status was examined again between December 2020 and February 2021 (2nd wave).

**Results:**

The seroprevalence of antibodies against SARS-CoV‑2 was 5.1% at the end of the study in February 2021. The cumulative incidence was 3.9% after a median observation period of 261 days.

**Conclusion:**

We observed a low risk of SARS-CoV‑2 infection comparable to that of the general population in the examined cohort of healthcare workers involved in the acute care of COVID‑19 patients under the applied hygiene and protective measures.

## Hintergrund

Medizinisches Personal, das in die Versorgung von COVID‑19-Patienten involviert ist, ist einem besonderen Risiko für eine Infektion mit SARS-CoV‑2 ausgesetzt [7, 9]. Insbesondere in Bereichen der Akutversorgung, wie Notfall- und Intensivmedizin, ist durch enge Kontakte sowie Hochrisikoprozeduren, wie z. B. Beatmungstherapie [4], das Risiko zusätzlich erhöht. Zudem sind oligo- oder asymptomatische Infektionen nicht immer von Beginn an zu identifizieren, was die Logistik effizienter Schutzmaßnahmen in der Notaufnahme erschwert.

Ziel dieser Studie war es, das Risiko einer Infektion mit SARS-CoV‑2 im zeitlichen Verlauf der Pandemie für Krankenhauspersonal anhand einer Kohorte von Mitarbeitern des Universitätsklinikums Regensburg zu evaluieren.

## Methodik

Einschlusskriterium war der regelmäßige Kontakt zu SARS-CoV-2-Patienten im Rahmen der Arbeitstätigkeit. Potenzielle Studienteilnehmer wurden in den betroffenen Bereichen mittels Informationsveranstaltungen über die Studie in Kenntnis gesetzt. Bei Studieneinschluss und im Abstand von jeweils 14 Tagen erfolgten venöse Blutentnahmen zur serologischen Untersuchung auf Antikörper gegen SARS-CoV‑2. Zudem wurde während der ersten Studienperiode Rachenspülwasser asserviert und bei Serokonversion auf eine Virusreplikation mittels PCR untersucht. Begleitend zu jeder Blutentnahme erfolgten eine tympanale Messung der Körpertemperatur sowie eine Befragung hinsichtlich subjektiven Wohlbefindens, des Auftretens von Symptomen eines viralen Atemwegsinfekts (Fieber, Husten, Kopf- oder Gliederschmerzen) sowie eines Kontakts zu COVID‑19-Patienten seit der letzten Blutentnahme.

Die letzten Blutentnahmen waren initial zum Ende der ersten COVID‑19-Welle für Juli 2020 geplant. Um den Einfluss der zweiten Welle auf die Serokonversionsrate abzuschätzen, wurden eine erneute Blutentnahme und Serologie von Ende Dezember 2020 bis Anfang Februar 2021 ergänzt. Kombinierter Endpunkt der Studie war das Auftreten einer Serokonversion oder eine mittels PCR nachgewiesene Infektion mit SARS-CoV‑2.

Der Beobachtungszeitraum für jeden Probanden war von Studieneinschluss bis zur letzten durchgeführten Blutentnahme bzw. bis zum Eintreten des Endpunkts. Ein Proband wurde bei unklarem Infektionsstatus nachträglich aus der Analyse ausgeschlossen, da sich rückblickend zeigte, dass bereits vor Studieneinschluss eine SARS-CoV-2-PCR-Diagnostik einmal grenzwertig positiv (im Bereich der Nachweisgrenze) und einmal negativ war, jedoch mehrfach keine Antikörper nachweisbar waren. Bei einem Probanden trat 40 Tage nach Ende des Beobachtungszeitraums eine symptomatische SARS-CoV-2-Infektion auf, was ebenfalls nicht mehr in der Auswertung berücksichtigt wurde, da der Beobachtungszeitraum bereits abgeschlossen war.

Alle Probanden erklärten sich nach ausführlicher Aufklärung schriftlich mit der Studienteilnahme einverstanden. Die Studie wurde von der Ethikkommission der Universität Regensburg genehmigt (Votum 20-1763-101).

Um die Rate an falsch-positiven serologischen Resultaten zu reduzieren, wurde ein 2‑stufiges Verfahren eingesetzt. Als primäres serologisches Verfahren (Suchtest) erfolgte die Bestimmung von Antikörpern gegen das Spike-Protein von SARS-CoV‑2 mittels ELISA [10]. Als sekundäres Verfahren (Bestätigungstest) wurden bei allen reaktiven Proben Antikörper gegen das Nukleoprotein (Elecsys Anti-SARS-CoV‑2, Roche Diagnostics GmbH, Mannheim, Deutschland) bestimmt. Nur ein reaktives Ergebnis beider Testverfahren wurde als Serokonversion gewertet. Zudem ermöglichte die Messung von Antikörpern gegen das Nukleoprotein die Bestimmung des serologischen Status bei bereits geimpften Studienteilnehmern, da sich Antikörper im Serum von Geimpften im Gegensatz zu Rekonvaleszentenserum nur gegen das Spike-Protein richten.

Die entnommenen Blutproben wurden bei 2000 g für 10 min zentrifugiert. Das gewonnene Serum wurde bis zur weiteren Verarbeitung bei −20 °C aufbewahrt.

Das Hygienekonzept des Universitätsklinikums Regensburg sah bei Kontakt zu bestätigten COVID‑19-Fällen bzw. Verdachtsfällen das Tragen einer persönlichen Schutzausrüstung bestehend aus Handschuhen, einer FFP-2-Maske, einem „face shield“ bzw. einer Schutzbrille sowie einem Schutzkittel vor. Es bestanden konkrete Handlungsanweisungen zum An- und Ablegen der persönlichen Schutzausrüstung sowie zu den Schritten der Händedesinfektion nach Ablegen der Schutzausrüstung. Die Versorgung mit persönlicher Schutzausrüstung war über den gesamten Studienzeitraum gesichert. Zudem bestand (und besteht) seit Pandemiebeginn im gesamten Klinikum eine Mundschutzpflicht für alle Mitarbeiter und Patienten (medizinischer Mund-Nasen-Schutz). Eine generelle Pflicht zum Tragen einer FFP2-Maske anstatt eines medizinischen Mund-Nasen-Schutzes bestand für das Personal auch in Hochrisikobereichen nicht. Des Weiteren sah das Hygienekonzept bei Verdachtsfällen aufgrund der frühzeitigen Verfügbarkeit von Schnelltest stets einen PCR-Test vor, SARS-CoV-2-Antigen-Schnelltests wurden nicht durchgeführt. Ab April 2020 wurde bei allen hospitalisierten Patienten (auch ohne respiratorische Symptomatik) ein Aufnahmescreening mittels regulärer PCR-Testung durchgeführt. Im Verlauf der Studie ergaben sich keine relevanten Veränderungen des Hygienekonzepts. Verdachtsfälle oder COVID‑19-Patienten wurden auf gesonderten Normal- und Intensivstationen behandelt. Bei erhöhtem Patientenaufkommen wurden weitere Isolationsbereiche auch auf anderen Intensivstationen geschaffen.

Die statistische Auswertung der Daten wurde mittels GraphPad Prism 9.1.0 (GraphPad Software, San Diego, USA) und R 4.0.5 [12] durchgeführt.

## Ergebnisse

Von März bis Mai 2020 wurden insgesamt 206 Mitarbeiter des Universitätsklinikums Regensburg in die Studie eingeschlossen. Ein Proband wurde von der Analyse ausgeschlossen (s. oben). 42 % (*n* = 87) der Studienteilnehmer waren männlich. Das Alter der Probanden bei Studieneinschluss lag bei 81 Probanden (39,5 %) zwischen 18 und 30 Jahren, bei 53 Probanden (25,9 %) zwischen 31 und 40 Jahren, bei 40 Probanden (19,5 %) zwischen 41 und 50 Jahren und bei 30 Probanden (14,6 %) zwischen 51 und 65 Jahren. Ein Proband war über 65 Jahre alt. 22,4 % (*n* = 46) der Studienteilnehmer waren nach eigenen Angaben zum Zeitpunkt des Studieneinschlusses aktive Raucher.

Insgesamt 138 (67,3 %) Teilnehmer waren Pflegekräfte, 47 (22,9 %) Ärzte und 20 Probanden (9,8 %) Angehörige anderer Berufsgruppen (Kardiotechniker, Assistenzpersonal, Reinigungskräfte). Bezogen auf Pflegepersonal und Ärzte entsprach die Kohorte ca. 7 % aller pflegerischen und ärztliche Mitarbeiter des Universitätsklinikums Regensburg. Der Großteil der Teilnehmer war auf der Intensivstation (*n* = 122, 59,5 %) oder in der Notaufnahme (*n* = 55, 26,8 %) beschäftigt. Weitere Einsatzbereiche waren die COVID‑19-Station (*n* = 6, 2,9 %), die Bronchoskopie (*n* = 6, 2,9 %) oder die Kardiotechnik (*n* = 10, 4,9 %).

136 der 205 untersuchten Probanden (66,3 %) absolvierten die finale Serologie zwischen dem 18. Dezember 2020 und 18. Februar 2021. Der mediane Beobachtungszeitraum betrug 261 Tage.

**Figure 1:**
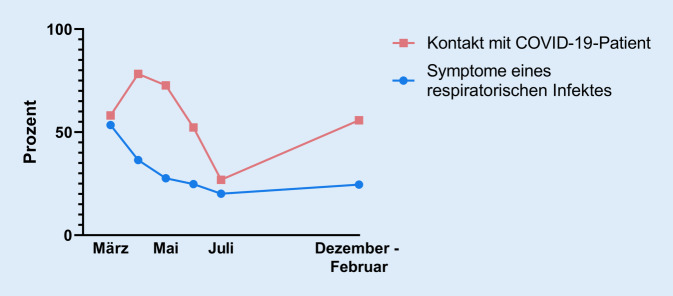

Die Häufigkeit von Symptomen eines respiratorischen Infekts zwischen 2 Blutentnahmen war über den Studienzeitraum abnehmend (Abb. 1). Zum Zeitpunkt der maximalen Anzahl an COVID‑19-Patienten am Universitätsklinikum Regensburg während der ersten Welle gaben 78,3 % und während der zweiten Welle 55,8 % der Teilnehmer an, Kontakt zu einem infektiösen Patienten gehabt zu haben (Abb. 1). Bei lediglich einer Körpertemperaturmessung wurde eine Temperatur über 38 °C festgestellt, wobei keine SARS-CoV-2-Infektion bei dem Probanden nachgewiesen werden konnte.

Während des Untersuchungszeitraums konnten bei 7 Probanden Antikörper gegen SARS-CoV‑2 im Screening und Bestätigungstest nachgewiesen werden, davon waren 2 Probanden asymptomatisch. Bei nur einem Probanden waren bereits Antikörper bei Studieneinschluss nachweisbar, wobei in dem korrespondierenden Rachenspülwasser eine Infektion mit SARS-CoV‑2 mittels PCR-Testung nachgewiesen werden konnte. Bei einer weiteren Serokonversion eines Probanden während der ersten Welle war die PCR-Diagnostik negativ. Der kombinierte Endpunkt aus Serokonversion oder nachgewiesener SARS-CoV-2-Infektion trat somit bei 7 Probanden auf. Die Inzidenzdichte des kombinierten Endpunkts betrug 0,062 Ereignisse pro Beobachtungsjahr bzw. 16,8 Infektionen pro 100.000 Personentage.

Bei 3 Infektionen im November 2020 bestand ein enger zeitlicher (innerhalb von 9 Tagen) und räumlicher Zusammenhang (gleicher Abteilungsbereich), sodass hier von einem Infektionscluster auszugehen ist.

**Figure 2:**
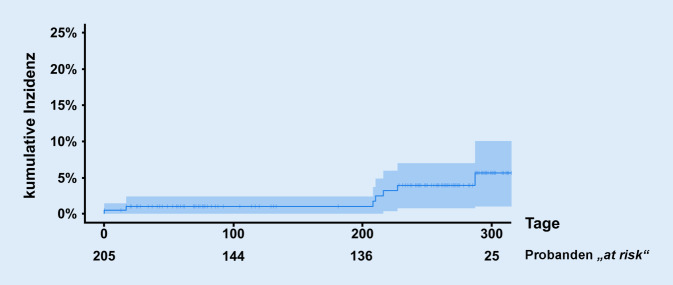

**Figure 3:**
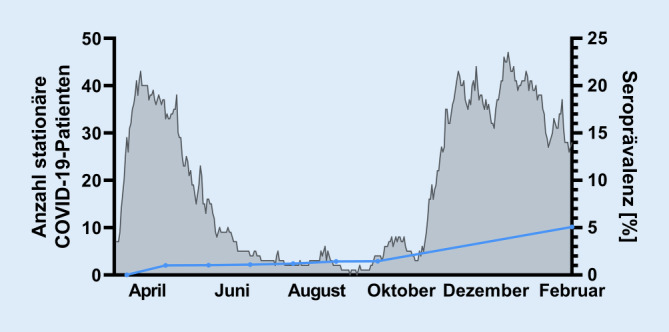

In einer Kaplan-Meier-Analyse betrug die kumulative Inzidenz zum Zeitpunkt der medianen Beobachtungsdauer (261 Tage seit Studieneinschluss) 3,9 % (95 %-Konfidenzintervall 0,7–7 %, *n* = 103 Probanden „at-risk“ verblieben, Abb. 2). Die kumulative Seroprävalenz für Antikörper gegen SARS-CoV‑2 betrug am Ende der ersten COVID‑19-Welle im Juli 2020 1,2 %. Zum Studienende betrug die kumulative Seroprävalenz bezogen auf die Anzahl an Probanden, bei denen die finale Serologie durchgeführt wurde, 5,1 % (Abb. 3).

**Figure 4:**
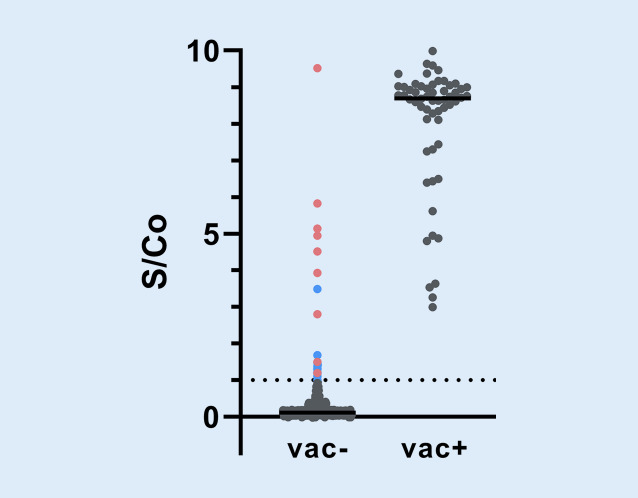

Insgesamt wurden zwischen März 2020 und Februar 2021 1223 serologische Untersuchung auf Antikörper gegen das Spike-Protein durchgeführt (Abb. 4). Bei den bereits geimpften Studienteilnehmern fanden sich im Durchschnitt höhere Signale zu Cut-off-Quotienten (S/Co) in der serologischen Messung gegen das Spike-Antigen als bei den Teilnehmern mit durchgemachter SARS-CoV-2-Infektion.

Die Studie wurde im Februar 2021 aufgrund der hohen Rate an bereits geimpften Teilnehmern beendet.

## Diskussion

In dieser Studie wurden 205 Klinikmitarbeiter mit möglichem Kontakt zu COVID‑19-Patienten über einen medianen Zeitraum von 261 Tagen während der ersten und zweiten COVID‑19-Welle hinsichtlich einer symptomatischen oder asymptomatischen Infektion mit SARS-CoV‑2 untersucht. Die kumulative Seroprävalenz für Antikörper gegen SARS-CoV‑2 betrug am Studienende im Februar 2021 5,1 %. Die kumulative Inzidenz einer Serokonversion oder symptomatischen Infektion betrug nach der medianen Beobachtungsdauer von 261 Tagen 3,9 % (Kaplan-Meier-Schätzer).

Bereits in mehreren Studien wurde das Risiko, sich mit SARS-CoV‑2 zu infizieren, für Mitarbeiter im Gesundheitswesen sowohl in Deutschland als auch international serologisch untersucht. Dabei fanden sich stark divergierende Ergebnisse. In mehreren deutschen Untersuchungen bewegte sich die Seroprävalenz für Mitarbeiter im Gesundheitswesen während oder nach der ersten COVID‑19-Welle zwischen 1,3 und 4,7 % [5, 8, 13]. Zwei Metaanalysen ergaben im europäischen Durchschnitt eine Seroprävalenz von 7,7–8,5 % unter Mitarbeitern im Gesundheitswesen, wobei die Prävalenz über den Verlauf der ersten Welle ansteigend war [2, 6]. Im Gegensatz hierzu fand sich in dieser Studie zum Ende der ersten Welle im Juli 2020 eine deutlich niedrigere Seroprävalenz von 1,2 %. Nicht nur im Vergleich zu anderen Studien von Mitarbeitern in Gesundheitswesen, sondern auch im Vergleich zu Populationsstudien ist dies eine niedrigere Seroprävalenz. So fand sich beispielsweise im Rahmen der KoCo19-Studie [11] in einer Stichprobe der Münchner Bevölkerung im Juni 2020 eine Seroprävalenz von 1,82 % und im November von 3,27 %.

Ein Grund für die in dieser Studie gefundene niedrige Seroprävalenz bei Klinikmitarbeitern könnte die konsequenten Umsetzung von Hygienemaßnahmen mit Isolation von bestätigten Fällen und Verdachtsfällen sein. Bereits zu Beginn der Pandemie wurden am Universitätsklinikum Regensburg Verdachtsfälle und bestätigte COVID‑19-Patienten konsequent isoliert. Eine vollständige persönliche Schutzausrüstung – bestehend aus FFP-2-Maske, „face shield“ bzw. Schutzbrille, Handschuhen und Schutzkittel – bei Kontakt zu Verdachts- und gesicherten Fällen war jederzeit gewährleistet. Die kumulative Inzidenz pro 100.000 Einwohner von Stadt und Landkreis Regensburg bewegten sich während des Untersuchungszeitraums leicht unterhalb des bayerischen Durschnitts. Aufgrund eines großen Einzugsgebiets für Mitarbeiter und Patienten sind jedoch die Fallzahlen der unmittelbaren Umgebung nur begrenzt aussagekräftig. Des Weiteren konnten durch die 2‑stufige Teststrategie mit einem Screeningtest (Spike-Protein) und einem Bestätigungstest (Nukleoprotein) falsch-positive Resultate weitestgehend vermieden werden. Auf 1223 Serologien wären ausgehend von der im Vorfeld bekannten Testspezifität von 99,3 % [10] 9 falsch-positive Ergebnisse zu erwarten gewesen, tatsächlich konnten 12 falsch-positive Testresultate durch die duale Teststrategie identifiziert werden.

Während einige Studien die Seroprävalenz als Punktprävalenz bestimmt haben [1, 8], zeigt diese Studie die Seroprävalenz über den Zeitraum der ersten und zweiten Pandemiewelle in Deutschland bis zum Beginn von Impfungen gegen SARS-CoV‑2 auf. In einer kürzlich publizierten prospektiven Kohortenstudie [3] mit 17.383 Teilnehmern von Juni bis Dezember 2020 fand sich eine höhere Inzidenzdichte von 57,3 Infektionen pro 100.000 Personentage im Vergleich zu dieser Studie (16,8 Infektionen pro 100.000 Personentage). Untersucht wurden Mitarbeiter im Gesundheitswesen in England, die bisher nicht an COVID‑19 erkrankt waren. Da jedoch die kumulative COVID‑19-Fallzahl im Vereinigten Königreich zum Ende des Studienzeitraums mit 36.770,98 Fällen pro 1 Mio. Einwohner deutlich höher war als in Deutschland (21.012,62 Fälle pro 1 Mio. Einwohner, COVID‑19 Data Repository, Johns Hopkins University), ist nicht von einem vergleichbarem Risiko auszugehen.

In Relation zu den positiven Testresultaten in Deutschland zum Ende der Studie von 2,85 % der Gesamtbevölkerung (RKI, Stand 18.02.2021) ist unter angewandten Schutzmaßnahmen von einem erfreulich niedrigen Infektionsrisiko für Krankenhausmitarbeiter auszugehen. Dies gilt insbesondere, da die hier untersuchte Kohorte sich größtenteils aus Mitarbeitern in Hochrisikobereichen (Intensivstation, Notaufnahme) zusammensetzte und dementsprechend eine deutlich erhöhte Exposition aufwies. Zudem handelt es sich bei den Infektionszahlen des RKI nur um Fälle mit positiver PCR-Diagnostik mit einer möglichen Dunkelziffer an nicht erfassten Infektionen.

## Limitationen

Als wichtige Limitation der Studie ist die geringe Anzahl an Probanden in Relation zur Gesamtanzahl der Mitarbeiter zu sehen. Auch war die Verteilung der Einsatzbereiche mit Fokus auf Notaufnahme und Intensivstation nur eingeschränkt repräsentativ für die Gesamtheit des Personals. Da das Risiko einer potenziellen SARS-CoV-2-Exposition von Mitarbeitern in diesen Bereichen höher als das der Grundgesamtheit des Personals ist, ist von einer potenziellen Verzerrung der Ergebnisse mit tendenzieller Überschätzung des Infektionsrisikos auszugehen. Des Weiteren war nur ein geringer Anteil der Studienteilnehmer im Bereich der COVID‑19-Normalstation eingesetzt. Die Aussagekraft bezüglich des Infektionsrisikos in diesem Bereich ist daher deutlich eingeschränkt. Zudem war nur bei 66,3 % der Studienteilnehmer ein vollständiges Follow-up möglich, was die Aussagekraft der Seroprävalenz einschränkt. Um diese zensierten Daten zu kompensieren, wurde jedoch mittels Kaplan-Meier-Schätzer die kumulative Inzidenz über den Beobachtungszeitraum berechnet. Aufgrund der insgesamt niedrigen Fallzahlen hatten die 3 mutmaßlich im Rahmen eines Clusters aufgetretenen SARS-CoV-2-Infektionen einen signifikanten Einfluss auf die Seroprävalenz und kumulative Inzidenz.

## Fazit

In dieser Studie konnte prospektiv anhand einer Kohorte von 205 Klinikmitarbeitern eines Universitätsklinikums gezeigt werden, dass am Ende der Untersuchungsperiode im Februar 2021 die Seroprävalenz für Antikörper gegen SARS-CoV‑2 bei 5,1 % lag. Die kumulative Inzidenz betrug nach 261 Tagen 3,9 %. Insgesamt ist bei angewandten Hygiene- und Schutzmaßnahmen ein nur gering erhöhtes Risiko für eine Infektion bei Klinikmitarbeitern in Relation zur Normalbevölkerung vorhanden.
